# Self-inflicted injury and the older trauma patient: a 20 year review of suicide attempts and outcomes

**DOI:** 10.1007/s41999-021-00561-w

**Published:** 2021-09-20

**Authors:** Kathryn B. Schaffer, Tala Dandan, Dunya Bayat, Matthew R. Castelo, Summer H. Reames, Linda Hutkin-Slade, Walter L. Biffl

**Affiliations:** Scripps Memorial Hospital Trauma Service, 9888 Genesee Ave., LJ601, La Jolla, CA 92037 USA

**Keywords:** Older, Trauma, Suicide, Intentional injury, Mental health

## Abstract

**Purpose:**

Older patients (Older) have complex health management needs often requiring additional resources. Mental health disorders are common among trauma patients, yet minimal information on older suicidal related injury and outcomes exists. A review of trauma patients with intentional self-inflicted injury at one trauma center was done to describe and identify unique elements of this cohort of patients.

**Methods:**

Trauma registry data from 2000 to 2019 were reviewed for intentional injury and data abstracted included demographics, injury severity, diagnoses, comorbidities and outcomes. Cohorts by age were compared: Older (65 +) vs Younger (< 65). Values considered significant at *p* ≤ 0.05.

**Results:**

557 suicide attempts were identified with 9% among Older patients. Most patients were male with median age of 75 years for Older and 35 years for Younger cohort, with similar length of stay (LOS) and injury severity scores (ISS). Penetrating injury was more common among Older patients with firearm used most often, 34% vs 14% for Younger. Differences were evident between male and female Older patients with ISS 16.7 vs 5, *p* < 0.01 and mortality, *p* = 0.03. The outcome of discharge to home was significantly different between Older and Younger, 6% vs 20% (*p* < 0.05). A difference in mortality was evident, Older 38% vs Younger 18% (*p* < 0.05).

**Conclusion:**

With the growing aging population, it is important to acknowledge the resultant increase in concomitant mental health issues and suicidality among older patients, where depression may be undiagnosed and untreated. Providing care within this cohort may reduce future attempts and lessen the burden on the health care system.

## Key summary points

**Aim:**

To describe a cohort of older trauma patients treated for injuries related to intentional injury over 20 years.

**Findings:**

Intentional injury among older patients is rare, but differences are evident when comparing older and younger survivors of suicide attempts. Differences with mortality method of self-inflicted injury, discharge to home and injury severity between genders were identified.

**Message:**

Although older patients are not the typical suicide attempt patient presenting at hospitals, their management and discharge planning are often complex and challenging due to undiagnosed mental health conditions, pre-existing comorbidities and difficulties with mental health resource options for this age group.

## Background

Suicide is a significant cause of mortality globally. The World Health Organization (WHO) estimates that every year, over 700,000 individuals die by suicide and accounted for 1.3% of all deaths worldwide in 2019, making it the 17th leading cause of death. Older adults, age 65 years + , are projected to comprise approximately 20% of the population by 2030 worldwide and, although this group is more often undiagnosed with psychiatric illness and attempt suicide less frequently than younger age groups, the risk remains and incidence continues to quietly increase [1]. In the United States, the per capita rate of suicide has been steadily increasing every decade, especially for those 65 years and older. As of 2018, Americans 65 years and older had a suicide rate (16.8/100,000), slightly higher than the general population (14.8/100,000). This rate only increases as a person ages, rising to 20.1/100,000 for those 85 years and older [2].

Traumatic injury can be described as severe physical injury, with sudden onset, requiring immediate medical attention. The forces and manner in which a physical injury occurs, mechanism, can further be categorized as blunt or penetrating. Blunt trauma is defined as any injury to the body caused by forceful impact (motor vehicle collisions, falls) and penetrating trauma caused by an object piercing the skin (stabbings, gunshot wounds). Traumatic injury affects all ages differently, often with more challenging management and less favorable outcomes among vulnerable populations, such as older adults where it is the 7th leading cause of death [3, 4]. Common injuries seen in older trauma patients are due to nonintentional mechanisms (falls, motor vehicle related injuries) and seldom due to intentional means (physical assaults or self-inflicted injury) [5, 6].

In 2005, two percent of all trauma center admissions were patients who had attempted suicide, “intentional self-harm events” [7]. Patients presenting to a hospital with injuries related to self-inflicted harm are not always treated by a trauma service. Drug overdose related events are more likely treated by the emergency department and more critically injured are admitted to a trauma center. Suicide attempts resulting in more severe injuries include jumping from heights (blunt trauma) or self-inflicted gun shootings and stabbings (penetrating trauma). Suicide attempts in California among those 60 years and older are the highest rates in the United States and range from 21.9 to 26.1/100,000. In 2019, among those age 85 or older, the rate was 21.3/100,000, exceeding the national rate of 20.1/100,000 [8]. In San Diego, California, eighth largest county in the USA, intentional injury events were highest among people ages 50 and older, with an average suicide rate of 22.9/100,000 [9].

With suicide rates increasing worldwide among young and old, there is growing interest in prevention and outreach. The most common risk factors for suicide include a diagnosis of depression, loneliness/living alone, functional/physical disability, and a history of self-harm or previous suicide attempt(s) [10–25]. Limited social connectedness and lack of social support were also found to be risk factors for suicide ideation [25–27]. An additional study involving older institutionalized males found that hopeless perceptions contributed most to suicide ideas when depressive psychopathology was present [28]. The supporting evidence is clear that there are many variables to consider when evaluating and treating this complex population. The most common method of suicide is the use of firearms, followed by hanging/suffocation, poisoning, jumping, and cutting/stabbing [29]. The most common method of suicide among males is the use of firearms, while females are more likely to use medication overdose [4, 29, 30]. Survivors of intentional injury are the population of focus in this research.

## Methods

A retrospective review of trauma registry data identified all suicide attempts over 20 years from January 2000 to December 2019 was done at a single American College of Surgeons (ACS) verified Level II adult trauma center. Trauma Centers in the United States are designated and verified every 3 years and can vary from state to state. There are five designated levels, which depend on their resources, capabilities, and number of patients admitted yearly [31]. Patients admitted to a Trauma Center meet specific criteria to be transported to a hospital that provides trauma care, including mechanism of injury, physiologic parameters, risk factors, and any additional anatomical injuries. This criterion helps Emergency Medical Services (EMS) determine which level of care their patient needs.

The study population included all trauma patients who presented with self-inflicted intentional injury mechanisms. Data were reviewed and cohorts were divided by age and designated for the purposes of this review as Younger (< 65 years) or Older (65 years and older). Suicide attempts transported to the hospital with minor injuries, not meeting trauma criteria, were seen by the Emergency Department (ED), and excluded from this study. Eligible patients were categorized in cohorts based on predominant mechanism of self-inflicted injury, blunt vs. penetrating. Trauma registry data were collected and analyzed.

Data elements abstracted included: demographics, mechanism of injury, diagnoses, complications, injury severity score (ISS), comorbidities, and outcomes. ISS calculations are generated by the trauma registry and based on injury diagnoses and AIS (Abbreviated Injury Scale) classification. The AIS is an internationally recognized anatomical scoring system correlating injury severity to patient outcomes and often used to describe traumatic injury. Patients with a psychiatric history were identified based on medical chart history. Mechanisms of injury, such as firearm use, were analyzed separately. Cohorts by age were identified: Older (65 + years) vs. Younger (< 65 years) and compared by injury severity, length of stay, injury mechanism and outcome.

Analysis of the study population was completed through descriptive statistics of means, medians, standard deviations (SD), interquartile ranges (IQR), and percentage scores where appropriate. A subgroup analysis was performed to determine the difference in outcome for patients based on age and gender. Comparisons were made using two proportion *Z*-tests, unpaired *T-*tests, Wilcoxon test, and Fisher’s Exact test. Statistical analyses utilized R version 3.6.1, GraphPad QuickCalcs and MEDCALC statistical software. *P* values were considered significant at *p* $\le$ 0.05.

## Results

Most suicide attempts arriving at our hospital are treated by the trauma service, with the exception of intentional drug overdose cases, normally treated by the emergency department medical staff. A cohort of 557 cases meeting study criteria was identified as trauma patients surviving a suicide attempt, meeting trauma triage criteria and transported to a designated trauma center for care over the 20-years period. 507 (91%) comprised the Younger cohort while Older patients comprised 9% of all suicide attempts (Table 1). Males were the predominant gender in both cohorts, comprising 71% of the Younger and 76% of Older (*p* = 0.49) patients. Although not statistically significant, mean age for male Older patients was 78.5 years compared 70 years for Older females, *p* = 0.14.

**Table 1:** Descriptive summary of intentional injury events over 20 years

2000–2019	Younger	Older	*p* value
	*n* = 507	*n* = 50	
Age, Mean (SD)	36 (13.4)	76.7 (8.7)	** < *0.05***
Gender, *n* (%)			
Male	362 (71)	38(76)	0.49
Injury Severity Score (ISS)			
Mean (SD)	13 (14.1)	14 (14.4)	0.21
Mechanism, *n* (%)			
Penetrating	313 (62)	40 (80)	< 0.05
Firearms use, *n* (%)			
Yes	71 (14)	17 (34)	< 0.05
Length of Hospital Stay (days)			
Mean (SD)	7 (11.5)	5.9 (9)	0.26
Pre-existing conditions, *n* (%)			
Psychiatric disorders	246 (49)	23 (46)	0.73
Physical disorders	78 (15)	10 (20)	0.39
No previous diagnosis	252 (50)	24 (48)	0.81
Outcome, *n* (%)			
Death	91 (18)	19 (38)	< 0.05
Psychiatric facility	221 (44)	16 (32)	0.11
Other acute care facility	47 (9)	4 (8)	0.97
Home, no assistance	103 (20)	3 (6)	< 0.05
Skilled nursing facility/rehab	27 (5)	4 (8)	0.64
Other	18 (4)	4 (8)	0.25

**Fig. 1:**
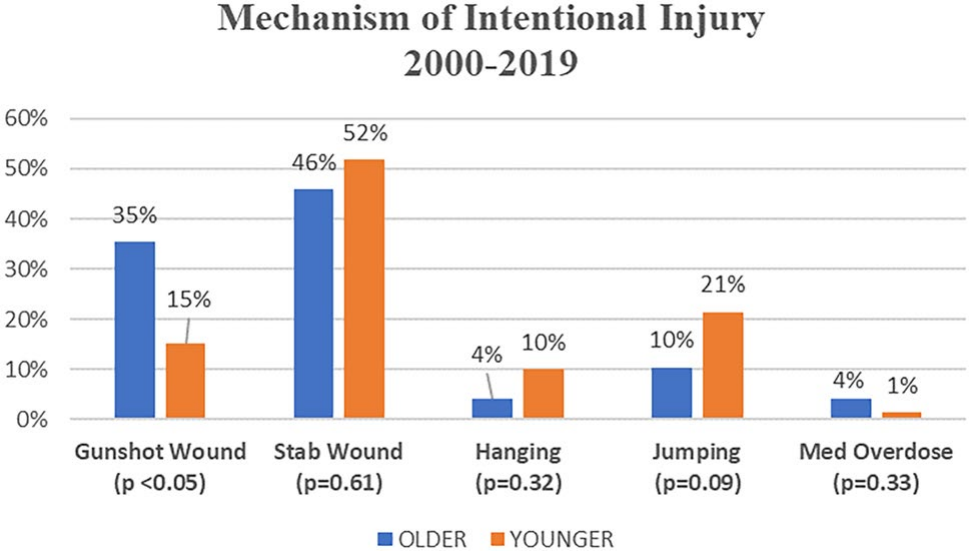
Mechanism of intentional injury Older vs. Younger

Mechanisms of suicide attempts varied and Older patients more often chose a penetrating mechanism, accounting for 80% of attempts in this cohort as compared to 62% among Younger patients. The most common mechanism among the Older cohort was firearm use and stabbings with sharp objects and the remaining 20% were blunt trauma mechanisms, predominantly jumps from heights. Among the Younger cohort there was a variety of intentional blunt mechanisms, including jumping from high levels, hangings, and medication overdose (Fig. 1). When comparing firearm use between Older and Younger, the Older group had a higher proportion of use and this difference was statistically significant, *p* < 0.05. Firearms were used in 17 (34%) of the Older cases, all but one being male patients. For these 17 firearm-related events, the mean age was 79 years and 14 of the 17 expired. 84% of male Older patients resorted to a penetrating mechanism of injury compared to 58% of the Older females, but this difference was not statistically significant, *p* = 0.13.

The range of injury severity scores (ISS) among all intentional injury events ranged from 2 to 23, with similar ranges for both Younger (ISS 2–20) and Older patients (ISS 2–23). Mean ISS was similar between Younger and Older (13 vs 14, *p* = 0.21), describing moderate severity as well as indicates these traumas were not multi-trauma injuries but more often involving a single body region. This is especially evident with penetrating mechanisms, whereas blunt trauma events (i.e., falls from heights) were more often polytrauma injury events. Head injury was the most common body region involved for both Younger and Older cohorts.

Proportions of medical comorbidities including pre-existing psychiatric disorders, did not differ between the two cohorts. Furthermore, nearly half of patients from both Younger and Older cohorts had no previous diagnosis of pre-existing psychiatric conditions. Among the Older patients, 53% of males had no history of a previous psychiatric diagnosis or suicidal ideation history as compared to 33% of the Older females. It must be noted that psychiatric history was often incomplete in the medical record, thus this observation may underestimate the true prevalence of a patient’s psychiatric history.

The length of hospital stay was also similar between Younger and Older cohorts, with a median hospital stay of 3 days for Younger vs 2 days for Older patients, *p* = 0.37. A larger proportion of the Younger patients were sent home (20%) compared to the Older patients (6%) (*p* < 0.05). There was no statistical difference among survivors in both cohorts who were discharged to a psychiatric facility, where about half of survivors from both cohorts were discharged to (32% vs 44%, *p* = 0.11). Furthermore, no difference between genders of those sent to a psychiatric facility was found for Younger and Older (*p* = 0.26). Although no gender differences were found when comparing all 557 patients, two statistically significant differences were evident between male and female older patients for ISS and outcome of death (Table 2).

**Table 2:** Descriptive summary of intentional injury among older adults by gender

2000–2019	Male	Female	*p* value
	*n* = 38	*n* = 12	
Age, Mean (SD)	77.9 (8.9)	73.1 (7.2)	0.09
Injury Severity Score (ISS)			
Median (Interquartile range)	17 (2, 25)	4 (1, 9)	0.02
Mechanism, *n* (%)			
Penetrating	32 (84)	7 (58)	0.13
Length of hospital stay (days)			
Median (Interquartile range)	1 (1, 6)	4 (1, 8.25)	0.45
Pre-existing conditions, *n* (%)			
Psychiatric disorders	15 (39)	8 (67)	0.09
Physical disorders	8 (21)	2 (17)	0.59
No previous diagnosis	20 (53)	4 (33)	0.24
Outcome, *n* (%)			
Death	18 (47)	1 (8)	0.03
Psychiatric facility	9 (24)	7 (58)	0.06
Other acute care facility	2 (5)	2 (17)	0.50
Home, No assistance	3 (8)	0	0.75
Skilled nursing facility/rehab	2 (5)	2 (17)	0.50
Other	4 (11)	0	0.57

Mortality among the Older patients was 38% and 18% for the Younger (*p* < 0.05). A majority of Older patients (79%) expired during the resuscitation phase in the emergency department shortly after arrival. The remaining patients died in the intensive care unit (ICU) and one in the operating room. The median time to death for the Older patient was 2 h after arrival and 8 h for the Younger deaths. Most deaths in the Younger cohort occurred after admission in the ICU and more than a third survived the initial resuscitation phase in the emergency department.

## Discussion

This retrospective review of one trauma center’s encounters with elderly trauma suicide attempts and subsequent management supports what has been reported in the literature with similar outcomes. In the Western United States, suicide is endemic and elderly survivors of intentional injury account for disproportionately higher morbidity and mortality. It has been known for some time that older patients, particularly males, are at a higher risk for suicide for many reasons, including isolation and lack of social support once their partner dies. These results support the well documented association between older males and more frequent use of penetrating mechanisms. We also know that among younger persons, for every 100 attempts, there is one completion and, for older adults, for every four attempts there is one completion [32].

The complexity of care associated with the inherent needs of these patients is challenging and potentially more so with elderly patients with pre-existing comorbidities. However, there was no significance difference evident when assessing pre-existing conditions among cohorts, which does not support the literature. Risk factors have been documented to be significant indicators for a person’s suicidality. Therefore, further examining these comorbidities might help explain the difference among these two cohorts. Given their complex comorbidities and diminished mental health, most Older patients (94%) either died or were sent to another facility for continued medical care (skilled nursing facility or inpatient rehabilitation). This is not surprising since these were trauma patients with severe injuries and complex medical management. These results were to be expected and support the literature examining older age in association with elevated mortality.

The lower percentage of older patients discharged to home is related to several issues. Oftentimes patients have been experiencing a decline in their ability to live independently prior to their attempt, or their attempt and subsequent treatment have left them even more debilitated. There is no safe discharge plan to home due to isolation, lack of familial support, or the patient’s partner may not be able to provide the appropriate level of care. These patients have less physical resilience than the younger patients and cannot “bounce back” at the same level.

Health care workers often encounter patients with injuries sustained from intentional self-harm first in the emergency or trauma service setting. The response and resources utilized for treatment first focus on the physical injuries and then the mental health needs, utilizing resources set up by the psychiatric response team and social workers. Adding advanced age to this combination of injury and mental illness creates complexity for management and discharge planning, different from the younger population with similar mechanism of injury. It is important to intervene as early as possible, focusing on the trajectory of depression and suicidal ideation and behavior. Continual screening should occur looking at life events, feelings of hopelessness, and psychopathology. This critical earlier detection can happen in the community, not just with primary physicians but also with friends, family, and community members. It should include working with all seniors to develop a personal crisis plan, personalized warning signs, self-management strategies, reasons for living, and emergency steps. There are a growing number of effective, evidence-based brief interventions that may be used to assist suicidal patients. One such program is Attempted Suicide Short Intervention Program (ASSIP), which can be offered while patients are in the hospital. This brief intervention therapy administered in conjunction with clinical treatment has shown to be a promising tool in reducing suicidal behavior over time and is one example of emerging interventions used today [33].

A variety of issues often impacts discharge to psychiatric facilities: lack of beds, funding, the severity of injury and the inability of the behavioral health unit to care for the patient, or simply the acuity of the suicidal ideation has lessened, and the patient no longer requires inpatient psychiatric care. For many patients, their active suicidal ideation passes within a few days to weeks. It is critical to get people through the intense suicidal ideation and work with them to develop a personal crisis plan with personalized warning signs, self-management strategies, reasons for living, and emergency steps, etc.

While not statistically significant, an additional unexpected finding was the longer average length of stay among the younger cohort. Compared to previous studies, the older population tends to have longer hospital stays, often related to pre-existing comorbidities. This finding could be explained by the common issue of lack of available beds in behavioral health units which leads to delay of discharge and extends LOS. While the Younger cohort had over 500 patients, the Older cohort consisted of 50 patients. This disproportionate sample size is a limitation of this investigation; however, the cohorts were large enough to support the conclusions made. The reason for the disproportionate sample sized in that the rates of older trauma patients being admitted for intentional injury is much lower than the rates for the younger population. Additionally, there is evidence from previous research that older patients are often successful with their suicide attempts, this small cohort was anticipated. A third limitation of this study was lack of post-discharge data for either cohort, which would have provided additional follow-up information on outcomes.

The use of larger data sources, such as registries and international databanks, would help explain the differences found among the younger and older patients. One study conducted by Crandall et al., used the National Trauma Data Bank (NTDB) to look at patients admitted for suicide attempts between 1995 and 2002 in the US. They found that, among the 1812 patients, the elderly suicide group had a higher mortality, were more likely to have a psychiatric history, and had a higher mean ISS compared to older trauma patients who presented to the hospital after motor vehicle collision (MVC). These patients were also less likely to be discharged home and had both longer ICU and ventilator days [34]. Additional resources that might support these findings include but are not limited to the National Electronic Surveillance System (NEISS), National Emergency Department Sample (NEDS), and National Hospital Ambulatory Medical Care Survey (NHAMCS). Utilization of these databases would provide larger sample sizes and potentially stronger correlations among the elderly population and intentional injuries.

Mental health support and treatment continue to be a growing challenge in managing patients with psychiatric conditions, often undiagnosed or untreated. Older patients may not telegraph their intentions, although anecdotally, loved ones may report they were sadder, more depressed, and withdrawn. There are many reasons older patients consider suicide. They are often concerned about being a burden to their family, worried about their loss of independence if they go to a different level of care, or that their quality of life is not what they want and they feel that they are done living, and they want to control when and how they die. Additionally, seeking mental health services is often a greater stigma in the older population than amongst younger people, where it is more common and sanctioned.

Although falls are the most common injury associated with older trauma, the rare incidence of intentional injury among the aging population should not be ignored. It is prudent that health care providers acknowledge mental health issues in older patients and recognize that their management and needs are different from the younger population inflicting self-harm. With 20% of suicide victims ultimately making another attempt within a year, one must address the underlying issues in this cohort to reduce the incidence of recidivism [7]. While the older suicidal population may not represent the majority of suicide attempts at our Level II trauma center, the increased mortality and complicated hospital course compared to the younger cohort suggest that healthcare facilities underestimate this growing issue.

Psychiatric follow up is imperative after a suicidal event, however, a psychiatric referral after an emergent trauma hospitalization is not sufficient in addressing the myriad of factors that can contribute to self-harm and suicidal ideation amongst older patients. Prevention would ideally occur at a primary care level to provide needed support and resources. Geriatric health care specialists are a growing specialty, along with older patient/elderly focused emergency and hospital stay care. Adding a level of mental health support for our older patients will help this need and support health care workers manage care and recovery for this growing vulnerable population at risk.

## Data Availability

Datasets will not be shared due to institutional restriction in data use agreement but data supporting the findings of this study are available from the authors upon reasonable request.
